# Hepatitis C virus core antigen, an earlier and stronger predictor on sustained virological response in patients with genotype 1 HCV infection

**DOI:** 10.1186/1471-230X-14-47

**Published:** 2014-03-13

**Authors:** Bo Feng, Rui-Feng Yang, Qing Xie, Jia Shang, Fan-Yun Kong, Hai-Ying Zhang, Hui-Ying Rao, Qian Jin, Xu Cong, Yun-Ye Liu, Yi Kang, Lai Wei

**Affiliations:** 1 Peking University People’s Hospital, Peking University Hepatology Institute, Beijing Key Laboratory of Hepatitis C and Immunotherapy for Liver Diseases, Beijing, PR China; 2 Department of Infectious Diseases, Ruijin Hospital, Shanghai Jiao Tong University School of Medicine, Shanghai, PR China; 3 Department of Infectious Diseases, Henan Provincial People’s Hospital, Zhengzhou, PR China

**Keywords:** Hepatitis C, HCV core antigen, Antiviral treatment, Interferon-α

## Abstract

**Background:**

Earlier kinetics of serum HCV core antigen (HCVcAg) and its predictive value on sustained virological response (SVR) were investigated in patients with genotype 1 HCV infection during antiviral treatment.

**Methods:**

In a multi-centered, randomized and positive drug-controlled phase IIb clinical trial on type Y peginterferon α-2b ( NCT01140997), forty-eight CHC patients who participated in pharmacokinetics were randomly divided into 4 cohorts and treated with PegIFNα (type Y peginterferon α-2b 90 μg, 135 μg, 180 μg and PegIFNα-2a 180 μg, respectively, once a week) and ribavirin (< 75 kg, 1000 mg daily and ≥ 75 kg, 1200 mg daily) for 48 weeks, and then followed up for 24 weeks. 32 patients infected with genotype 1 HCV and completed the whole process were included in this study. HCV RNAs were detected at baseline, and weeks 4, 12, 24, 48 and 72 using Cobas TaqMan. ARCHITECT HCVcAg was performed at 24, 48, 72, 96, 120 and 144 h in addition to the above time points. The receiver operating curves (ROCs) were performed to study the predictive values of HCVcAg decline on SVR.

**Results:**

Following antiviral treatment, serum HCVcAg levels rapidly declined within the first week and correlated well with corresponding HCV RNA at baseline, weeks 4, 12, 24, 48 and 72 (*r*_*s*_ = 0.969, 0.928, 0.999, 0.983, 0.985 and 0.946, respectively, *P* < 0.001). All of the areas under the receiver operating curves (AUROCs) were more than 0.80 and showed good predictive power on SVR at 24, 48, 72, 96, 120 and 144 h. The144 h was the best predictive time point of HCVcAg decline on SVR because of its largest AUROC (more than 0.90).

**Conclusions:**

Early kinetics of serum HCVcAg predicts SVR very well in genotype 1 CHC patients during antiviral treatment, and its reduction value at 144 h is an earlier and stronger predictor on SVR than rapid virological response and early virological response. (TRN: NCT01140997).

## Background

In the past 10 years, treatment response of chronic hepatitis C (CHC) has been increasingly improved based on the combination of pegylated interferon (PegIFN) α and ribavirin. The standard of care for newly diagnosed patients with hepatitis C virus (HCV), administered for 24 or 48 weeks, yielded sustained virological response (SVR) in approximately 80% and 40-50% of patients infected with HCV genotypes 2–3 and 1, respectively [1]. However, about 50% genotype 1 chronic hepatitis C patients, who are the most common worldwide, do not achieve SVR. Additionally, besides expensive costs, almost all patients treated with PegIFNα and ribavirin experience one or more adverse events during the course of therapy [2]. As such, it is very important to predict the virological response patterns before and during anti-viral therapy.

Many factors, including virus, host and drugs, are associated with the response to IFN-α-based therapy. Initial viral kinetics is most helpful in predicting treatment outcome. Patients who achieved a rapid virological response (RVR), which is defined as undetectable HCV RNA by Week 4 of therapy, are very likely to achieve SVR. By contrast, those who still have detectable HCV RNA levels by Week 12 of therapy have a very low likelihood of obtaining SVR [3,4]. As an illustration of the point which early viral responses are influenced by all pretreatment factors, once treatment has started, the IL28B genotype loses predictive value for SVR [5,6]. Treatment duration should be tailored to the virological response at weeks 4 and 12, and eventually week 24. Treatment for all HCV genotypes should be stopped at week 12, if the HCV RNA decline is less than 2 log10 IU/ml and at week 24, if HCV RNA is still detectable (≥50 IU/ml) [7]. That is to say, some patients do not know whether they need stopping antiviral treatment or keep going until week 24. Additionally, positive predictive value (PPV) of RVR is high, but its negative predictive value (NPV) is low. Marcellin et al. found that SVR can be achieved in 88% of patients with undetectable HCV RNA and 43% of those with detectable HCV RNA at week 4 [8]. Therefore, it is very important to search for a new marker for that will predict SVR earlier and more accurately.

Quantitative detection of HCV core antigen (HCVcAg) may be an alternative, which was reported to confirm viral replication in hepatitis C infected patients [9,10]. Several studies showed that levels of serum HCVcAg correlate with those of HCV RNA in CHC patients [11-13]. In acute hepatitis C cases, HCVcAg can pick up a great majority of HCV RNA positive samples [14], and closely track HCV RNA dynamics throughout the course of the disease. Moreover, a sharp and parallel decrease of HCV RNA dynamics was observed during treatment with antiviral drugs 3 months after onset [15]. In the current study, based on a phase IIb clinical trial that examined the safety and efficacy of type Y pegylated interferon alfa-2b (NCT01140997, Pegabin®, Tebao Pharmaceuticals Inc., China) in CHC patients, we characterized dynamic changes of HCVcAg levels in patients with genotype 1 HCV infection during antiviral treatment and investigated the predictive value of earlier kinetics of serum HCVcAg on SVR.

## Methods

### Study design

The clinical trial, based by the current study, was multi-centered, randomized, open-labeled and used a positive drug-control on 210 naïve patients with HCV infection. The patients were randomly divided into 4 cohorts and treated with PegIFNα (type Y PegIFN α-2b 90 μg, 135 μg, 180 μg and PegIFNα-2a 180 μg, respectively, once a week) and ribavirin (< 75 kg, 1000 mg daily and ≥ 75 kg, 1200 mg daily) for 48 weeks, and then followed up for 24 weeks. Among all 48 CHC patients who participated in pharmacokinetics, only those infected with genotype 1 HCV and who completed the whole process were included in this study.

### Inclusion and exclusion criteria

The patients ranged in age from 18 to 65 years old, had positive anti-HCV and HCV RNA levels of greater than 2000 IU/ml for at least six months, and also provided evidence of written informed consent. The study was approved by the ethical committees of Peking University People’s Hospital, Peking University First Hospital, Beijing Friendship Hospital, 302 Hospital of People’s Liberation Army, Beijing Youan Hospital, First Affiliated Hospital of Jilin University, Central-south University Xiangya Hospital, Sichuan University West China Hospital, Chongqing Medical University Second Affiliated Hospital, Fuzhou Infectious Disease Hospital, Guangzhou Eighth People’s Hospital, Nangfang Hospital, the First Affiliated Hospital of Guangxi Medical Universtiy, the Second Affiliated Hospital of Harbin Medical University, the First Affiliated Hospital of Anhui Medical University, Jinan Infectious Disease Hospital, the First Affiliated Hospital of Lanzhou University, the First Affiliated Hospital of Nanchang University, 81 Military Hospital, Jiangsu Provincial People’s Hospital, the Second Hospital of Nanjing, Changhai Hospital of Shanghai, Shanghai Public Health Clinical Center, Renji Hospital of Shanghai, 85 Hospital of People’s Liberation Army, Ruijin Hospital of Shanghai, Huashan Hospital of Shanghai, Shenzhen Third People’s Hospital, the Third Affiliated Hospital of Hebei Medical University, the First Affiliated Hospital of Shanxi University, Tianjin Third Central Hospital, the First Affiliated Hospital of Wenzhou Medical College, Huazhong Science and Technology University Tongji Hospital, Tangdu Hospital, the First Affiliated Hospital of Zhengzhou University and Henan Provincial People’s Hospital. The study protocol conformed to the ethical guidelines of the 1975 Declaration of Helsinki, and was performed according to the guidelines of the International Conference on Harmonization for Good Clinical Practice. Exclusion criteria included significantly abnormal liver function (for example total bilirubin levels greater than 2 ULN, albumin levels lower than 35 g/L, PTA levels lower than 60% or evidence of decompensated liver disease and hepatocarcinoma), pregnancy or inability to practice adequate contraception, significant systemic or major illnesses other than liver disease, preexisting lower blood cells (white blood cell levels lower than 3 × 10^9^/L, absolute neutrocyte counts lower than 1.5 × 10^9^/L, platelet levels lower than 90 × 10^9^/L and hemoglobin lower than lower limit of normal (LLN) or known history of antiviral or immunosuppressive therapy, and evidence of other viruses infection such as HAV, HBV, HEV, HIV, EBV and CMV. Liver injury caused by other agents was excluded, for example alcohol, drug, auto-immunity and metabolic abnormality among others.

### Lab examination

HCV RNA levels were measured at baseline, at weeks 4, 12, 24, 48 and 72 of therapy. The COBAS AmpliPrep/COBAS TaqMan automated real-time polymerase chain reaction (PCR) platform (Roche Molecular Systems, Pleasanton, CA) was used. This assay has a lower limit of detection (LLOD) of 15 IU/mL and a lower limit of quantification of 43 IU/mL.

The ARCHITECT HCVcAg (Abbott Diagnostics, Wiesbaden, Germany), which is a quantitative chemiluminescent microparticle immunoassay and run on the fully automated ARCHITECT instrument, was used to quantify HCVcAg. As the manufacturer instructed, the assay was performed and HCVcAg levels lower than 3.0 fmol/l were considered nonreactive. ∆HCVcAg was defined as a log10 reduction of serum HCVcAg levels between other time points and baseline.

### Definitions

SVR was defined as an undectable HCV RNA level (<50 IU/ml), 24 weeks after cessation of treatment. RVR was defined as undetectable HCV RNA in a sensitive assay (lower limit of detection ≤ 50 IU/ml) at week 4 of therapy and maintained up to the end of treatment [7]. The SVR corresponds to a cure of infection in > 99% of cases [16]. ∆HCVcAg was defined as a log10 reduction of serum HCVcAg levels between other time points and baseline.

### Statistical analysis

The statistical significance of differences between groups was analyzed by the student’s *t*-test, one-way ANOVA, Fisher’s exact test or the Mann–Whitney *U*-test. Correlation between HCVcAg and HCV RNA levels was measured using the Spearman’s correlation coefficient. The optimal predictive values of ∆HCVcAg and HCVcAg at different time points were assessed by calculating the areas under the univariate receiver operating characteristics (AUROC) curve. Sensitivity, specificity, PPV, and NPV were analyzed to determine the reliability of predictors of the response to therapy. All statistical analyses were performed with SPSS 16.0 (Chicago, IL, USA). Two-tailed *P* values less than 0.05 were considered significant statistically.

## Results

### Baseline characteristics of sample

All thirty-two patients included in this study were infected with genotype 1b HCV. There were 16 male and 16 female patients. The mean age was 45.16 ± 11.06. Mean baseline viral load and HCV antigen levels were 6.33 ± 0.88 log10 IU/mL and 3.66 ± 0.49 log10 fmol/L, respectively. Based on the outcomes, these patients were divided into SVR and non-SVR groups, of which the baseline characteristics are described below (Table 1). There were no differences between SVR and non-SVR groups in baseline parameters including gender, ALT, HCV RNA, HCVcAg, dose and category of PegIFNα and excluding age.

**Table 1:** Baseline patient characteristics for patients with sustained virological response (SVR) and non-sustained virological response (N-SVR)

**Parameters**	**SVR**	**N-SVR**	***P***
Male/female	10/6	6/10	0.325
Age (years)	41.90 ± 11.08	51.36 ± 8.31	0.019
ALT (U/L)	47.38 ± 3.65	47.27 ± 6.87	0.988
HCV RNA (log10 IU/mL)	6.39 ± 0.57	6.21 ± 1.14	0.557
HCVcAg (log10 fmol/L)	3.61 ± 0.54	3.75 ± 0.36	0.441
No. of each cohorts^a^	6/3/7/5	2/6/2/1	0.057

^a^The numbers of patients in each cohorts (Y PegIFN α-2b 90 μg, 135 μg, 180 μg, and PegIFNα-2a 180 μg).

### Dynamic changes of HCVcAg levels during antiviral treatment

**Figure 1:**
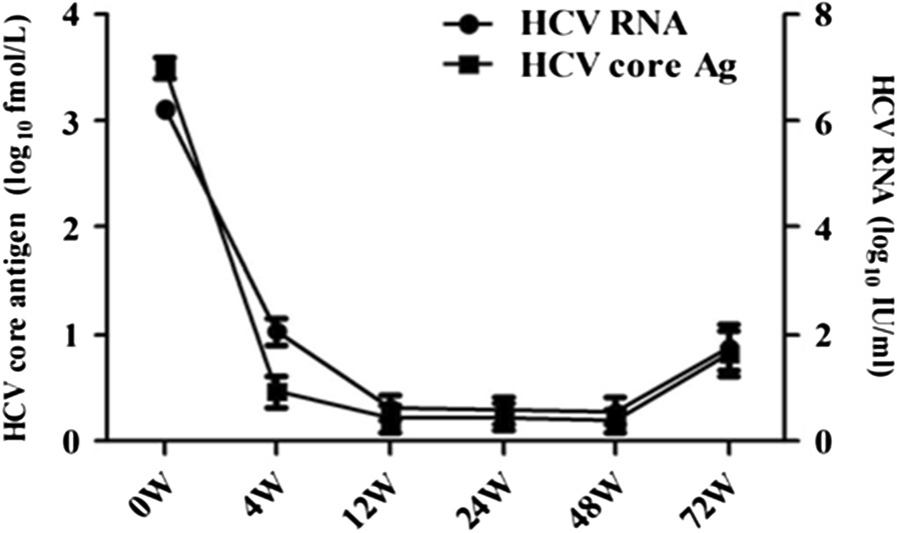
**Dynamic changes of HCVcAg and HCV RNA levels during antiviral treatment.** Following antiviral treatment, serum HCVcAg levels closely track those of HCV RNA and rapidly declined within the first 4 weeks.

Following antiviral treatment, serum HCVcAg levels closely track those of HCV RNA and rapidly declined within the first 4 weeks (Figure 1). At baseline, week 4, 8, 12, 24, 48 and 72, HCVcAg levels showed excellent correlation with HCV RNA; spearman correlation coefficients were 0.956, 0.937, 0.999, 0.983, 0.995 and 0.980, respectively (*P* < 0.001).

### Predictive values of ∆HCVcAg on SVR at various time points of the first 12 weeks of treatment

**Figure 2:**
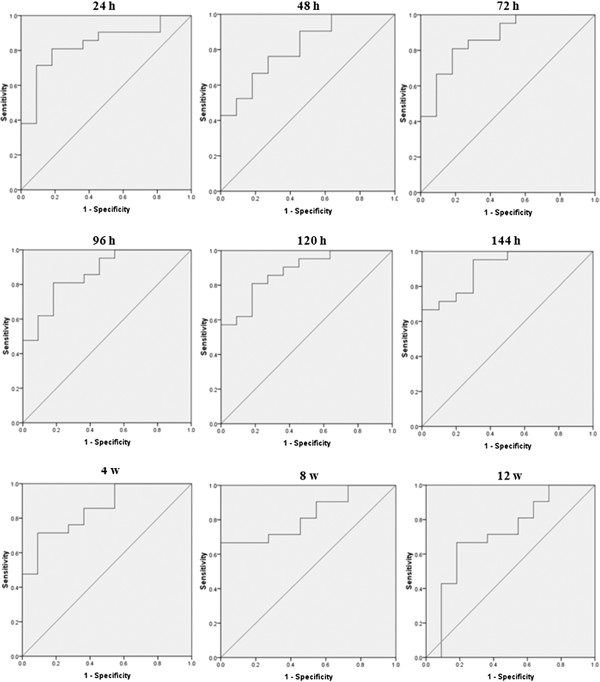
**ROCs of predictive values of ∆HCVcAg at various time points of the first 12 weeks on SVR.** AUROCs calculated were 0.835, 0.814, 0.870, 0.866, 0.879, 0.913, 0.853, 0.823 and 0.719 at 24 h, 48 h, 72 h, 96 h, 120 h, 144 h, and weeks 4, 8, 12.

To investigate the predictive values of ∆HCVcAg at various time points within the first 12 weeks on SVR and to search for the best cutoff values, ROCs were performed (Figure 2). Based on these ROCs, important parameters were worked out including AUROC, the optimal cutoff values, sensitivity, specificity, PPV and NPV (Table 2). AUROCs were 0.835, 0.814, 0.870, 0.866, 0.879, 0.913, 0.853, 0.823 and 0.719 at 24 h, 48 h, 72 h, 96 h, 120 h and 144 h, and at weeks 4, 8 and 12. The highest AUROC was 0.913 at 144 h. At 24 h, 48 h, 72 h, 96 h, 120 h and 144 h, and at week 4, cutoff values, which predicted SVR, were 0.785, 1.090, 1.034, 1.262, 1.138, 1.096 and 3.231; the corresponding sensitivity was 0.810, 0.762, 0.810, 0.810, 0.818, 0.762 and 0.714; specificity was 0.818, 0.727, 0.818, 0.818, 0.818, 0.800 and 0.909; PPVs were 1.000, 1.000, 1.000, 1.000, 1.000, 1.000 and 1.000; and NPVs were 0.846, 0.846, 0.846, 0.846, 0.846, 0.769 and 0.688, respectively. While PPVs were similar across time points, NPVs differed. The optimal combination of AUROC, PPV and NPV, with a cutoff value of 0.976 was at 144 h, with AUROC 0.913, PPV of 0.913 and NPV 1.000.

**Table 2:** Area under the ROC curve (AUROC), sensitivity, specificity, and predictive values of sustained virological response based on a log 10 decrease in total HCV core antigen, across cutpoints and timepoints after initial antiviral treatment

	**24 h**	**48 h**	**72 h**	**96 h**	**120 h**	**144 h - 1**	**144 h - 2**	**4 w**	**8 w**	**12 w**
AUROC	0.835	0.814	0.870	0.866	0.879	0.913	0.913	0.853	0.823	0.719
(95% CI)	(0.691-0.980)	(0.663-0.965)	(0.742-0.998)	(0.738-0.994)	(0.760-0.998)	(0.799-1.000)	(0.799-1.000)	(0.720-0.985)	(0.680-0.965)	(0.520-0.917)
Cutoff^a^	0.785	1.090	1.034	1.262	1.138	0.976	1.096	3.231	3.780	3.770
Sensitivity	0.810	0.762	0.810	0.810	0.810	0.952	0.762	0.714	0.667	0.667
Specificity	0.818	0.727	0.818	0.818	0.818	0.700	0.800	0.909	1.000	0.818
PPV	1.000	1.000	1.000	1.000	1.000	0.913	1.000	1.000	1.000	1.000
NPV	0.846	0.846	0.846	0.846	0.846	1.000	0.769	0.687	0.611	0.687

AUROC, areas under the univariate receiver operating characteristic curve; PPV, positive predictive value; NPV, negative predictive value.

^a^Cutoff was defined as change of log 10 fmol/L.

### Predictive power of HCVcAg on SVR at various time points of the first week of the treatment

Predictive values of HCVcAg on SVR were measured using by AUROCs at 24 h, 48 h, 72 h, 96 h, 120 h and 144 h. The corresponding AUROCs were 0.236, 0.225, 0.195, 0.186, 0.182 and 0.126 at these various time points, respectively. This showed that the absolute values of HCVcAg levels had no predictive power on SVR in genotype 1 HCV infected patients.

### Predictive value of HCV RNA decline at week 4 of the treatment on SVR

**Figure 3:**
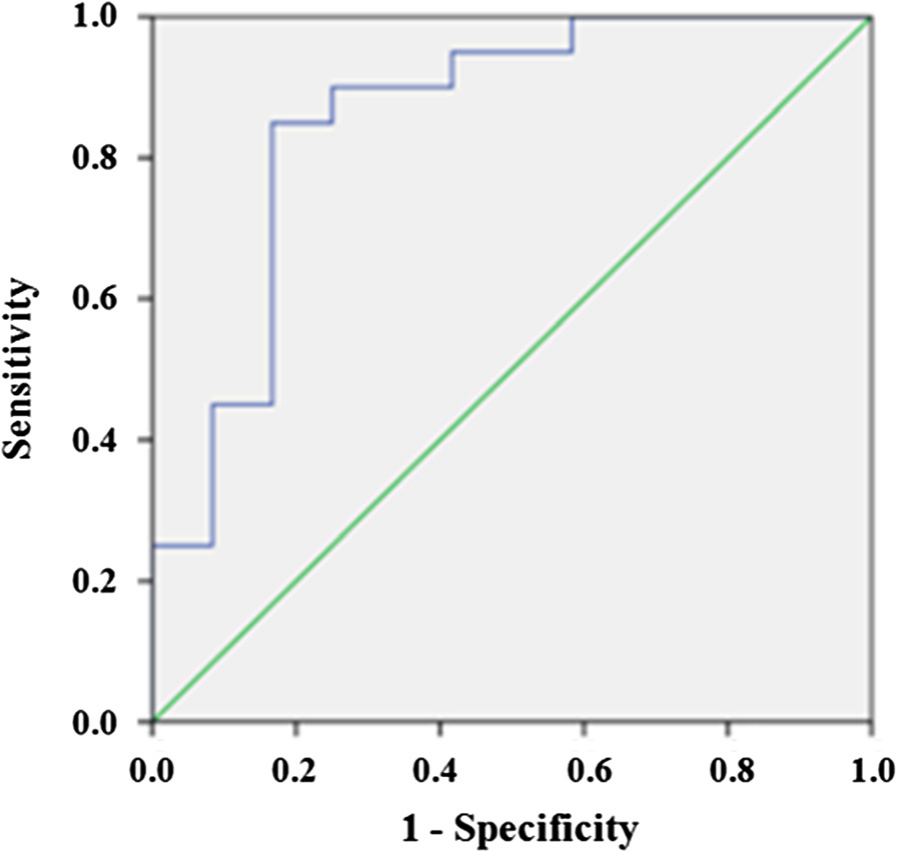
**ROC curves of predictive values of HCV RNA at week 4 on sustained virological response (SVR).** When the optimal cutoff value was 3.770, the corresponding AUROC was 0.854 (95% CI 0.705-1.000).

ROC was performed on the basis of the relationship between ∆HCV RNA at week 4 and SVR (Figure 3). When the cutoff value was 3.770, the corresponding AUROC, sensitivity, specificity, PPV and NPV were 0.854 (95% CI 0.705-1.003), 0.850, 0.833, 1.000 and 0.706. Calculated sensitivity, specificity, accuracy, PPV and NPV of RVR were 0.229, 1.000, 0.500, 1.000 and 0.407. Table 3 showed the results comparing the predictive value of ∆HCVcAg at 144 h, and weeks 4, 8, and 12 to that of ∆HCV RNA at week 4. Results indicated comparable PPVs, both 100%, but higher NPV (76.9%) and higher AUROC (0.913) for HCVcAg at 144 h compared to HCV RNA at week 4 (70.6%, 0.854) respectively.

**Table 3:** Predictive values and Area under the ROC curve (AUROC) of sustained virological response based on a log 10 decrease in total HCV core antigen and HCV RNA concentrations after initial antiviral treatment

	**PPV**	**NPV**	**AUROC (95% CI)**
∆HCVcAg (log10 fmol/L)			
144 h	100%	76.9%	0.913 (0.799-1.000)
Week 4	100%	68.7%	0.853 (0.720-0.985)
Week 8	100%	61.1%	0.823 (0.680-0.965)
Week 12	100%	68.7%	0.719 (0.520-0.917)
∆HCV RNA (log10 IU/mL)			
Week 4	100%	70.6%	0.854 (0.705-1.000)

PPV, positive predictive value; NPV, negative predictive value; AUROC, areas under the univariate receiver operating characteristic curve.

### Comparison of predictive power between a log_10_ decrease in HCVcAg at 144 h and RVR/EVR

As for the 32 patients included in this study, the parameters of predictive value of a log_10_ decrease in HCVcAg at 144 h, RVR and early virological response (EVR) were calculated (Table 4). RVR had higher specificity (1.000) and PPV (1.000), and EVR has higher sensitivity (1.000) and NPV (1.000). Conversely, sensitivity and NPV of RVR, and specificity and PPV of EVR were lower. The accuracy of RVR and EVR was lower than a log_10_ decrease of HCVcAg at 144 h.

**Table 4:** **Comparison of predictive power of a log**_**10**_**decrease in HCVcAg at 144 h and rapid virological response (RVR)/early virological response (EVR)**

	**Sensitivity**	**Specificity**	**Accuracy**	**PPV**	**NPV**
∆HCV Ag ≥ 1 log	0.952	0.727	0.875	0.870	0.889
RVR	0.286	1.000	0.531	1.000	0.423
EVR	1.000	0.273	0.750	0.724	1.000

### Influence of baseline parameters on ∆HCVcAg at 144 h

**Figure 4:**
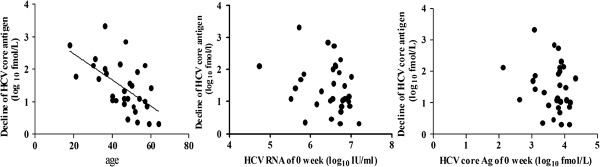
**Influence of baseline parameters on ∆HCVcAg at 144 h.** Decline of HCVcAg at 144 h showed a correlation with age (*r*_*s*_ = -0.583, *P* < 0.001), and no correlation with baseline viral loads and HCVcAg levels (*P* = 0.107 and *P* = 0.288, respectively).

As indicated in Figure 4, a decline of HCVcAg at 144 h showed a correlation with age (*r*_*s*_ = -0.583, *P* < 0.001), and no correlation with baseline viral loads and HCVcAg levels (*P* = 0.107 and *P* = 0.288, respectively). Additionally, gender, dose and category of PegIFNα had no effect on ∆HCVcAg at 144 h (*P* = 0.276, *P* = 0.458 and *P* = 0.623, respectively).

## Discussion

Several assays are used in the screening, diagnosis and management of HCV infection: detection of specific antibody to HCV and HCV RNA, and HCV genotyping [17,18]. So far, the early dynamic change of HCV nucleic acid levels, which are direct proof of viral replication, is a very important marker in predicting antiviral response patterns in CHC patients. As a gold standard, the kinetics of HCV RNA has been widely used in response-guided and individual therapy. Conjeevaram et al. thought that viral kinetics provide a summative reflection of the baseline factors, including insulin resistance, gender, age and genetic polymorphisms, which are involved in transducing the response to these agents [19]. However, as described above, nucleic acid quantitative detection of LOD of 50 IU/mL has some limitations, especially in developing countries.

The first assay for HCVcAg was developed in 1999 [20]. Now sensitivity and specificity of quantitative HCVcAg assay has been increasingly improved. The Architect HCVcAg assay (Abbott Diagnostics), with a dynamic range of quantification (3.0 – 20,000 fmol/L), is commercially available. It shows a good correlation with HCV RNA in various CHC populations including HCV monoinfection, HCV/HIV and HCV/HBV coinfection, and can reflect viral replication as HCV RNA does [21]. Here, similar changes of HCVcAg and HCV RNA were observed during PegIFNα combined with ribavirin treatment in genotype 1 HCV infected patients. Good correlations were showed between serum HCVcAg and HCV RNA levels at various time points observed in this study. Moreover, serum HCVcAg levels declined sharply in the first 4 weeks after treatment was initiated.

By calculating the AUROCs, we assessed the optimal predictive values of ∆HCVcAg at 24, 48, 72, 96, 120 and 144 h on SVR. We found that all of AUROCs were more than 0.80 and showed good predictive power at various time points in the first week. The second injection was performed at 168 h. We selected 144 h, which had the largest AUROC (0.913) as the best predictive time point. On the other hand, for obtaining the highest sensitivity and NPV, the lowest cutoff was obtained at quartile 0.25. Conversely, for obtaining the highest specificity and PPV, the highest cutoff was obtained at quartile 0.75. So, we selected 0.976 and 1.096 as cutoff values of 144 h, which both had a corresponding NPV and PPV of 1.000. That is, at the 144 h time point it will be unlikely that patients will have a ∆HCVcAg lower than 0.976 and those patients with a ∆HCVcAg of more than 1.096 will be likely to achieve SVR. On the other hand, if the ∆HCVcAg at 144 h is between 0.976 and 1.096, the corresponding NPV and PPV will be at least 0.769 and 0.913, respectively.

HCVcAg and HCV RNA have been shown to have a strong correlation (*r* = 0.86), from a sample where plasma HCV antigen was detected in 51 of 54 patients with an interpolated LOD cut off between 10^3^ and 10^4^ RNA IU/mL [22]. Chevaliez et al. thought the current assays for detecting HCVcAg are not suitable for monitoring changes of HCV replication power during antiviral therapy and response-guided therapy [10]. However, the hypothesis has never been proven in clinical practice. In the current study, we analyzed the predictive power of ∆HCV RNA at week 4 and RVR on SVR by ROC. The AUROC of ∆HCV RNA at week 4 was lower than that of ∆HCVcAg at 144 h. RVR which accuracy was 50% had higher PPV and lower NPV. We thought that the sensitivity of HCVcAg detection may be lower than that of HCV RNA when confirming viral replication and active infection, but HCVcAg may actually be more meaningful in reflecting dynamic changes of viral replication and in predicting SVR earlier during double treatment. If proven true by larger patient samples, HCVcAg detection will be more promising in clinical practice because it is less expense and easier to perform, especially in areas with limited resources.

As we well know, many baseline parameters including virus, host and drug factors are associated with response to IFN-α-based therapy. In the current study, we found that patient gender, dose and category of PegIFNα, baseline viral loads and HCVcAg levels had no correlation with ∆HCVcAg at 144 h, except for age. Further investigation should be performed to determine whether it is true or limited by numbers of patients. On the other hand, we did not differentiate null responders and relapsers because of limited receivers.

## Conclusions

Serum HCVcAg is a good marker for reflecting dynamic changes of HCV viremia in genotype 1 CHC patients during PegIFNα when combined with ribavirin treatment. Following antiviral treatment, serum HCVcAg levels rapidly declined within the first week similar to HCV RNA. All of the AUROCs were more than 0.80 and showed good predictive power on SVR at 24, 48, 72, 96, 120 and 144 h. 144 h was selected as the best predictive time point because of its largest AUROC (more than 0.90). 0.976 and 1.096 were selected as cutoff values of 144 h; both have a corresponding NPV and PPV of 1.000. Moreover, the predictive value of ∆HCVcAg at 144 h is better than that of ∆HCV RNA and negative HCV RNA at week 4.

## Abbreviations

CHC: Chronic hepatitis C; PegIFN: Pegylated interferon; HCV: Hepatitis C virus; SVR: Sustained virological response; RVR: Rapid virological response; PPV: Positive predictive value; NPV: Negative predictive value; HCVcAg: HCV core antigen; LLN: Lower limit of normal; HAV: Hepatitis A virus; HBV: Hepatitis B virus; HEV: Hepatitis E virus; HIV: Human immunodeficiency virus; EBV: Epstein-Barr virus; CMV: Cytomegalovirus; PCR: Polymerase chain reaction; LLOD: Lower limit of detection; AUROC: Areas under the univariate receiver operating characteristic curve.

## Competing interests

This study was sponsored in part by Xiamen Amoytop Biotech Co., Ltd.

## Authors’ contributions

BF and RFY contributed to study design, experimental process, data acquisition, statistical analysis and drafted the manuscript. FYK and HYZ contributed to experimental process, data acquisition, and statistical analysis. YK contributed to experimental process and drafted the manuscript. HYR and YYL participated in study planning and statistical analysis. QJ and XC contributed to study design, data analysis, and drafted the manuscript. LW, QX and JS participated in design, funding analysis and manuscript drafting. All authors read and approved the final manuscript.

## Pre-publication history

The pre-publication history for this paper can be accessed here:

http://www.biomedcentral.com/1471-230X/14/47/prepub
